# Clinical experience of whole-body computed tomography as the initial evaluation tool after extracorporeal cardiopulmonary resuscitation in patients of out-of-hospital cardiac arrest

**DOI:** 10.1186/s13049-020-00746-5

**Published:** 2020-06-11

**Authors:** Kelvin Jeason Yang, Chih-Hsien Wang, Yu-Cheng Huang, Li-Jung Tseng, Yih-Sharng Chen, Hsi-Yu Yu

**Affiliations:** 1 Department of Surgery, National Taiwan University Hospital, College of Medicine, National Taiwan University, No. 7, Chung-Shan South Road, Taipei, 100 Taiwan; 2 Department of Radiology, National Taiwan University Hospital, College of Medicine, National Taiwan University, Taipei, Taiwan

**Keywords:** Extracorporeal membrane oxygenation, Cardiopulmonary resuscitation, Extracorporeal cardiopulmonary resuscitation, Out-of-hospital cardiac arrest, Computed tomography, Hypoxic brain damage

## Abstract

**Background:**

The routine application of whole-body CT after extracorporeal cardiopulmonary resuscitation (ECPR) in out-of-hospital cardiac arrest (OHCA) has not been extensively investigated. We aimed to evaluate the benefit of CT in this context.

**Methods:**

We retrospectively analyzed all OHCA patients who had received ECPR between January 2006 to May 2019. Electronic records were reviewed to filter out patients who had a whole-body CT as their first clinical evaluation after ECPR. CT findings and major hospital outcomes were evaluated.

**Results:**

From January 2006 to May 2019, 700 patients had received ECPR in our institution. We identified 93 OHCA patients who received whole-body CT as the first clinical evaluation after ECPR. 22.6% of those had no acute findings detected on CT requiring immediate treatment. In the remaining 77.4%, CT had findings that might lead to alterations in clinical course. Most important findings were myocardial infarction (57.0%), hypoxic brain injury (29.0%), sternal/rib fractures (16.1%), aortic dissection (7.5%), pulmonary embolism (5.4%), and cardiac tamponade (5.4%). There were no significant differences in ICU/hospitalization days, time on ECMO support, survival and neurological outcomes between those with and without immediate CT. In our OHCA cohort, there were 27 patients with CT evidence of hypoxic brain injury, of whom 22.2% (n = 2) managed to wean from ECMO support, 14.8% (n = 4) survived to discharge, but only 3.7% (n = 1) survived with good neurological outcome. Hypoxic brain injury on CT has a 95% specificity in predicting poor neurological outcome, with a false positive rate of only 3.7%. Logistic regression suggested a potential correlation between CT findings of hypoxic brain injury and poor neurological outcome [Odds ratio (OR) = 12.53 (1.55 to 10.1), *p* = 0.02)].

**Conclusions:**

Routine whole-body CT after ECPR in OHCA patients appears to have a limited role, as the majority is caused by ACS. However, it may be a useful tool when CPR-related injury or non-ACS causes of OHCA are suspected, as well as in cases where the cause of OHCA is unknown. On the contrary, routine brain CT may be a valuable tool in guiding anticoagulant therapy during ECMO and in aiding outcome prediction.

## Introduction

According to the American Heart Association (AHA), there were more than 350,000 cases of out-of-hospital cardiac arrests (OHCA) in the United States in 2016, with a survival to discharge rate of only 11.4% [1]. Despite the remarkable advances in the field of resuscitation medicine, there has been limited improvement in the survival rate of OHCA patients. The use of extracorporeal membrane oxygenation (ECMO) in OHCA patient refractory to conventional cardiopulmonary resuscitation (CPR) has significantly increased the survival rate to as high as 38.7% [2–4]. The caseload of extracorporeal cardiopulmonary resuscitation (ECPR) is expected to increase continuously.

Clinical and post-mortem studies have demonstrated that acute coronary syndrome (ACS) is the most common cause of OHCA [5], however, studies have also shown that non-ACS causes account for approximately 22–34% of OHCA cases [5], which are potentially treatable if early diagnosis and treatment are achieved.

Computed tomography (CT) is a fast, cost-effective and widely available imaging modality. Recent guidelines of the European Resuscitation Council (ERC) recommend that CT scan may be performed to identify respiratory or neurological causes of OHCA, which enables prompt medical intervention [6]. However, the routine use of whole-body CT after return of spontaneous circulation (ROSC) in OHCA has not been extensively investigated and the diagnostic values remain uncertain, especially after ECPR. Over the last decade, there has been a gradual change in clinical practice in our institution. In the past, CT was mainly used as a negative selecting tool to identify those with poor prognosis (e.g. irreversible brain damage), but more recently it is used as a screening tool for other non-ACS causes for OHCA such as aortic dissection or pulmonary embolism. Therefore, this study was conducted to investigate the value of early whole-body CT after ECPR in OHCA patient.

## Materials and methods

Our institution started its ECMO program in 1994 and established a task force committee for ECPR back in 2003 [3]. The task force was responsible for the collection of data and maintenance of our ECMO database. All data were prospectively collected and analyzed on a regular basis. This study was approved by the institutional review board of National Taiwan University Hospital (NTUH – 201,810,079 RIN) and the board waived the need for informed consent.

In the current study, we selectively analysed the data from our ECMO database of all adult patients (age≧18) who had received ECPR following OHCA between January 2006 and May 2019. We excluded all patients whose index ECPR was performed in another institution because of uncertainty in arrest time, duration of CPR, and unstandardized ECMO management protocol. The patients whose OHCA was unwitnessed were also excluded, as well as those with incomplete follow-up data.

OHCA was defined as an event of cardiac arrest that occurred outside the hospital. For those patients who experienced OHCA, our certified emergency medical team performed CPR and defibrillation in compliance with the Advanced Cardiac Life Support guideline (ACLS), while being transported to our institution. Upon arrival at the Emergency room, the ECMO team was consulted to determine if the patient was eligible for ECPR. The contraindications, equipment and management protocol for ECMO have been described in our previously published studies [3, 4, 7]. The principle components of the ECMO circuit in our institution consisted of a centrifugal pump and a membranous oxygenator (Medtronic, Anaheim, CA, USA; Medos, Stolberg, Germany; Maquet, Rastatt, Germany). The unprimed circuit was pre-assembled and stored in a mobile cart. Prior to its use, the circuit was primed with normal saline containing 2 U/ml of heparin sulfate.

Patient demographics, baseline health and initial lab data were collected immediately after the set-up of ECMO. Additional information such as low flow duration (LFD) was also recorded (defined as the interval from the start of CPR to the initiation of ECMO support). Data on major outcomes and adverse events were also collected prospectively during hospitalization. Our primary outcome was a favorable neurological outcome at discharge, which was defined as a cerebral performance category (CPC) score of 1 or 2.

After the set-up of ECMO circuit, the patients would then undergo the appropriate investigation to determine the cause of their arrest at the clinician’s discretion. Most of the patients would either be transferred straight to the cardiac catheterization room for immediate coronary angiography (with or without primary coronary intervention) or to the radiology department for whole-body CT. For those who received immediate CT after ECPR, the radiologist would inject the contrast agent and perform the image according to a modified CT protocol based on previous studies [8–11]. The modified protocol is provided in the supplementary material (Additional file 1).

Retrospective review of the medical records was carried out to identify patients who had received whole-body contrast CT as their first investigation after ECPR. The following data were then collected: 1. Date / time in which the CT scan was performed 2. Findings of the CT scan based on the primary radiology report. The original CT images were independently reviewed by a board certified radiologist for validation. Any discrepancy in CT findings was reconciled by a third radiologist with experience in interpreting CT images of ECMO patients. For the purpose of clarification, the diagnosis of acute myocardial infarction on CT was made when a regional myocardial perfusion defect was observed, whereas the diagnosis of hypoxic brain injury was made when features such as loss of grey-white matter differentiation and cerebral edema with effacement of cerebral sulci were present on brain CT (Additional files 2 and 3: Supplemental Figure 1 and 2). To assess the diagnostic power of CT in detecting AMI and poor neurological outcome, coronary angiographic findings and a CPC score≧3 at discharge were used as gold standards.

### Statistical analysis

Baseline patient demographics were displayed either as mean ± standard deviation (SD) or percentage (%). Normally distributed continuous variables were displayed as mean ± SD and were analyzed using student *T* test. Continuous variables with a skewed distribution were presented as median and 25–75% interquartile range and were analyzed using the Mann-Whitney *U* test or Wilcoxon tests. Categorical variables were analyzed using the chi-square test. For those patients who received immediate CT, logistic regression analysis was performed. Independent prognostic variables affecting the major outcome (poor neurological status at hospital discharge i.e. CPC score 3 ~ 5) were first examined in univariate analysis. Variables were selected to enter the multivariable analysis if P value was less than 0.1. A P value of < 0.05 was considered statistically significant. The diagnostic power of whole body CT were presented as sensitivity, specificity, True/false positive, True/false negative, positive likelihood ratio (LR+) and negative likelihood ratio (LR-). All statistical analysis were performed using MedCalc statistical software version 19.1 (MedCalc Software, Ostend, Belgium).

## Results

**Fig. 1:**
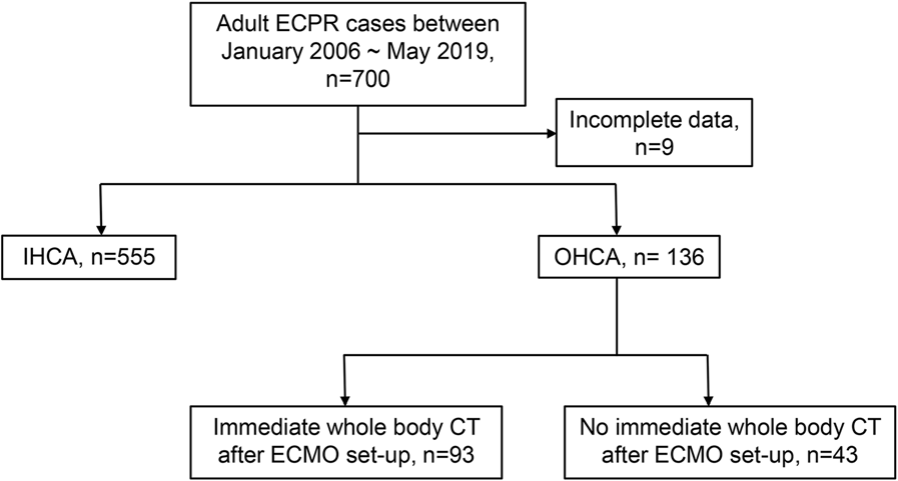
Flowchart of study patients. ECPR = Extracorporeal cardiopulmonary resuscitation, IHCA = In-hospital cardiac arrest, OHCA = Out-of-hospital cardiac arrest, ECMO = Extracorporeal membrane oxygenation

Between January 2006 and May 2019, a total of 700 patients experienced cardiac arrest and received ECPR in our institution. Among them, 555 were in-hospital cardiac arrest (IHCA) patients and 9 had incomplete follow-up data, thus were excluded from our study. The remaining 136 patients who had OHCA were included and further divided into two groups depending on whether they had received whole-body CT scan as their first evaluation tool after ECPR. Ninety-three patients were classified as the immediate CT group, and the remaining 43 patients as non-immediate CT group (Fig. 1).

The baseline demographic data of the two patient groups are summarized in Table 1. In brief, the immediate CT group consisted of mostly male patients and the average time interval between the set-up of ECMO to CT was 69.1 min. Those who received immediate CT exhibited a significantly lower incidence of late stage chronic kidney disease (CKD stage 4 / 5) but there was no significant difference in baseline serum creatinine level or estimated glomerular filtration rate (eGFR). In addition, the LFD in the immediate CT group was considerably longer when comparing to the non-immediate CT group (75.4 ± 129.9 and 45.0 ± 19.4 min, respectively, p = 0.03). There were no significant differences in age, comorbidities, Charlson’s comorbidity index (CCI), first documented rhythm and the cause of CPR between the two groups. In both groups, approximately 62% of CPR were related to acute coronary syndrome.

**Table 1:** Study population demographics

Demographic features	All, ***N*** = 136	Immediate CT group, ***N*** = 93	Non immediate CT group, ***N*** = 43	***P*** value
Male Gender, n (%)	115 (84.6)	84 (90.3)	31 (72.1)	0.006
Female Gender, n (%)	21 (15.4)	9 (9.7)	12 (27.9)	
Age (year)				
Mean ± SD	53.94 ± 12.7	54.7 ± 12.2	52.3 ± 13.8	0.31
Median (IQR)	53.85 (20.0–78.8)	54.4 (20–78)	53.4 (23–78)	0.43
Age > 60 years, n (%)	46 (33.8)	34 (36.6)	12 (27.9)	0.33
Age > 75 years, n (%)	4 (2.9)	3 (3.3)	1 (2.3)	0.78
Comorbidities, n (%)				
Diabetes mellitus	41 (30.2)	26 (28.0)	15 (34.9)	0.42
Hypertension	70 (51.5)	47 (50.5)	23 (53.5)	0.75
Liver cirrhosis	4 (2.9)	3 (3.2)	1 (2.3)	0.77
Coronary artery disease	46 (33.8)	32 (34.4)	14 (32.6)	0.83
Peripheral arterial disease	8 (5.9)	3 (3.2)	5 (11.6)	0.05
NYHA class 3/4	34 (25)	22 (23.7)	12 (27.9)	0.60
COPD	4 (2.9)	1 (1.1)	3 (7.0)	0.06
CKD	19 (14.0)	8 (8.6)	11 (25.6)	0.008*
CKD stage 4/ 5	10 (7.4)	4 (4.3)	6 (14.0)	0.05*
CKD on dialysis	8 (5.9)	4 (4.3)	4 (9.3)	0.25
Stroke	11 (8.1)	6 (6.5)	5 (11.6)	0.31
Causes of cardiac arrest, n (%)				
Acute myocardial infarction	85 (62.5)	58 (62.4)	27 (62.8)	0.96
Chronic heart failure	10 (7.4)	6 (6.5)	4 (9.3)	0.56
Dissecting aortic aneurysm	4 (2.9)	3 (3.2)	1 (2.3)	0.77
Cerebral hemorrhage	2 (1.5)	2 (2.2)	0 (0)	0.33
Pulmonary embolism	3 (2.2)	3 (3.2)	0 (0)	0.24
Arrhythmias	9 (6.6)	6 (6.5)	3 (7.0)	0.91
Acute myocarditis	2 (1.5)	0 (0)	2 (4.7)	0.04
Cardiac tamponade	1 (0.7)	1 (1.1)	0 (0)	0.50
Respiratory failure	1 (0.7)	1 (1.1)	0 (0)	0.50
Others	19 (14.0)	13 (14.0)	6 (14.0)	1.00
Initial Lab data (mean ± SD)				
Creatinine (mg/dL)	1.96 ± 2.08	1.92 ± 1.81	2.06 ± 2.60	0.73
eGFR (mL/min/1.73m^2^)	68.7 ± 41.7	64.7 ± 30.7	77.7 ± 59.3	0.10
Lactate (mmol/L)	13.0 ± 5.76	12.9 ± 5.72	13.3 ± 5.94	0.74
Charlson’s Comorbidity Index (CCI)				
Mean ± SD	3.94 ± 2.71	3.68 ± 2.55	4.51 ± 3.02	0.10
Median (IQR)	3.0 (2.0–5.0)	3.0 (2.0–5.0)	4.0 (3.0–6.0)	0.15
Low flow duration (min)				
Mean ± SD	65.9 ± 108.7	75.4 ± 129.9	45.4 ± 19.3	0.03*
Median (IQR)	49.5 (38.0–60.0)	53 (41.0–62.5)	42.0 (32.0–54.3)	0.004*
First Documented Rhythm, n (%)				
Pulseless VT / VF	90 (66.2)	61 (65.6)	29 (67.4)	0.83
PEA / Asystole	46 (33.8)	32 (34.4)	14 (32.6)	
ECMO to CT time (min)				
Mean ± SD	N/A	69.1 ± 24.84	N/A	N/A
Median (IQR)	N/A	65.0 (53.99 to 82.24)	N/A	N/A

*SD* standard deviation, *IQR* Interquartile range, *NYHA* New York Heart Association, *COPD* Chronic obstructive pulmonary disease, *CKD* Chronic kidney disease, *eGFR* estimated glomerular filtration rate, *VT* Ventricular tachycardia, *VF* Ventricular fibrillation, *PEA* pulseless electrical activity

Among the 93 patients who received immediate CT after ECPR, 21 (22.6%) had no acute findings requiring immediate treatment. On the other hand, 72 (77.4%) had findings that might result in alterations in clinical course. The major acute CT findings are displayed in Table 2**.** The most common finding was AMI, which was found in almost up to 60% of our patient. However, there were other diagnosis other than AMI that might require medical attention or even immediate intervention, including hypoxic brain injury (29%), acute aortic dissection (7.5%), pulmonary embolism (5.4%), cerebral hemorrhage/infarction (8.6%), pneumothorax (9.7%), cardiac tamponade (5.4%), and sternal/rib fractures (16.1%).

**Table 2:** Findings of whole-body computed tomography performed after extracorporeal cardiopulmonary resuscitation in out-of-hospital cardiac arrest patients

***N*** = 93	Incidence of CT findings
Acute myocardial infarction	53 (57.0%)
Hypoxic brain	27 (29.0%)
Cerebral hemorrhage	4 (4.3%)
Cerebral Infarction	4 (4.3%)
Pulmonary embolism	5 (5.4%)
Dissecting aortic aneurysm	7 (7.5%)
Cardiac tamponade	5 (5.4%)
Pulmonary consolidation / infiltration	77 (82.8%)
Atelectasis	19 (20.4%)
Pneumothorax	9 (9.7%)
Pleural effusion	19 (20.4%)
Bowel ischemia	5 (5.4%)
Sternal / rib fractures	15 (16.1%)

For patients who had findings of AMI on CT, the diagnostic accuracy was evaluated by correlating the results of subsequent coronary angiography, the results are displayed in Table 3. Of the 53 patients who had findings of regional myocardial perfusion defect on CT, 47/53 (88.7%) had compatible coronary lesions confirmed by coronary angiography. Whereas for those who had no regional myocardial perfusion defect on CT, 29/40 (72.5%) had normal coronary angiography. The sensitivity and specificity of CT in detecting possible AMI were 81.0 and 82.9%, respectively. The LR+ was 4.7 and LR- was 0.229.

**Table 3:** Diagnostic strength of computed tomography in detecting acute myocardial infarction and predicting poor neurological outcome

Major CT findings, n (%)	Hypoxic brain injury	Acute myocardial infarction
Number (%)	27/93 (29.0%)	53/93 (57.0%)
Outcome measured	Poor neurological outcome (CPC 3/4/5)	Positive coronary angiography
Number of patients with / without the associated outcome	71/22	58/35
True positive	26/27 (96.3%)	47/53 (88.7%)
False positive	1/27 (3.7%)	6/53 (11.3%)
True negative	21/66 (31.8%)	29/40 (72.5%)
False negative	45/66 (68.2%)	11/40 (27.5%)
Sensitivity	26/71 (36.6%)	47/58 (81.0%)
Specificity	21/22 (95.5%)	29/35 (82.9%)
Positive likelihood ratio (LR+)	8.06	4.7
Negative likelihood ratio (LR-)	0.66	0.229

*CPC* cerebral performance category

As briefly mentioned above, 29% of our OHCA cohort had evidence of hypoxic brain injury on CT after ECPR. We analyzed the CPC score of our patients and correlated with their CT findings (Table 3**)**. Of the 27 patients who had a CT showing features of hypoxic brain injury, 26/27 (96.3%) had a poor neurological outcome (CPC 3, 4 or 5) at discharge, with a reasonably low false positive rate of only 3.7%. However, when the brain CT was normal, only 21/66 (31.8%) had a favorable neurological outcome (CPC 1 or 2) at discharge with a false negative rate of 68.2%. The sensitivity and specificity of CT in detecting poor neurological outcome were 36.6 and 95.5%, respectively. The positive likelihood ratio (LR+) was 8.06 and the negative likelihood ratio (LR-) was 0.657.

The major hospital outcomes of all patients are shown in Table 4**.** Overall, the median duration of ECMO support was 3.0 days, and 50.7% of our OHCA patients weaned off ECMO and up to 36.8% were alive at discharge. Of those who survived at discharge, 70% had a favorable neurological outcome (CPC 1 or 2). When comparing the immediate CT group and the non-immediate CT group, no significant difference in major outcomes was observed.

**Table 4:** Outcomes of extracorporeal cardiopulmonary resuscitation in out-of-hospital cardiac arrest

Outcomes	All (***n*** = 136)	Immediate CT group (***n*** = 93)	Non-immediate CT group (***n*** = 43)	***P*** value
Ventilator days	11.6 ± 18.5	11.6 ± 19.6	11.7 ± 16.2	P = 0.98
ECMO days	3.3 ± 3.3	3.5 ± 3.8	3.0 ± 1.9	P = 0.30
ICU days	11.6 ± 16.9	10.8 ± 12.5	13.5 ± 24.0	P = 0.50
Hospitalization Days	20.4 ± 30.3	19.6 ± 28.3	22.2 ± 34.6	P = 0.65
Die on ECMO, n (%)	67 (49.2%)	47 (50.5%)	20 (46.5%)	P = 0.66
Weaned off ECMO and die, n (%)	19 (14.0%)	13 (14.0%)	6 (14.0%)	P = 1.00
Survival on discharge, n (%)	50 (36.8%)	33 (35.5%)	17 (39.5%)	P = 0.65
CPC 1/2 on discharge, n (%)	35 (25.7%)	23 (24.7%)	12 (27.9%)	P = 0.69

*ECMO* Extracorporeal membrane oxygenation, *CPC* Cerebral performance category

For patients who received immediate CT after ECPR, their respective outcomes based on CT diagnosis were analyzed and are displayed separately in supplemental materials (Additional file 4: Supplemental Table 1)**.** We found that the AMI group exhibited the highest ECMO weaning rate (52.8%), survival rate (39.6%) and rate of favorable neurological outcomes (26.4%) among all groups. On the contrary, when hypoxic brain injury is present on CT, ECMO weaning rate and rate of survival at discharge were only 22.2 and 14.8%, respectively. In addition, only 3.7% of the patient had favorable neurological outcomes at hospital discharge. For both cardiac tamponade and dissecting aortic aneurysm groups, there was no survival at discharge.

Risk factor analysis for predictive factors in poor neurological outcomes (CPC 3, 4 or 5) is presented in Table 5. Univariate analysis showed that high baseline serum creatinine level (*p* = 0.02), and a CT diagnosis of hypoxic brain injury (*p* = 0.001) were potential predictive risk factors for poor neurological outcome after ECPR. Whereas dialysis during hospitalization showed a trend of significance (*p* = 0.08). Notably, age / male gender / CCI / low flow duration / initial lactate level were not risk factors for poor neurological outcome. Results from the multi-variable logistic regression analysis revealed that CT diagnosis of hypoxic brain injury correlated with poor neurological outcome [Odds ratio (OR) = 12.53 (1.55 to 10.1), *p* = 0.02].

**Table 5:** Logistic regression analysis for poor neurological outcome at discharge in patients who received early CT

	Univariate		Multivariate	
	OR (95% CI)	*P*-value	OR (95% CI)	*P*-value
Age	0.98 (0.95 to 1.03)	0.44		
Age > 75	1.0 (0.10 to 10.05)	0.99		
CCI	1.11 (0.91 to 1.36)	0.28		
Male gender	1.11 (0.21 to 5.79)	0.90		
Low flow duration (min)	1.00 (0.99 to 1.03)	0.95		
Low flow duration > 60 min	1.27 (0.41 to 4.0)	0.68		
ECMO to CT time (min)	1.01 (0.99 to 1.03)	0.60		
Hypoxic brain injury on CT	12.4 (1.58 to 9.77)	0.001*	12.53 (1.55 to 10.1)	0.02*
Dialysis	2.40 (0.87 to 6.61)	0.08	2.14 (0.72 to 6.36)	0.18
Initial serum creatinine	2.22 (0.85 to 5.76)	0.02*	2.06 (0.72 to 5.91)	0.17
Initial eGFR	0.99 (0.98 to 1.01)	0.61		
Initial lactate level	0.99 (0.91 to 1.08)	0.82		

*OR* Odds ratio, *CI* Confidence interval, *CCI* Charlson’s comorbidity index, *ECMO* Extracorporeal membrane oxygenation, *CT* computed tomography, *eGFR* estimated glomerular filtration rate

## Discussion

Our study was initiated to evaluate the value of early diagnostic CT in OHCA patients following ECPR. Since ACS is the most common cause of OHCA, current guideline on post-resuscitation care recommends immediate cardiac catheterization if the cause of arrest was highly suspected of myocardial ischemia [12]. However, a number of clinical studies have demonstrated that a proportion of OHCA were caused by etiologies other than ACS or even mimic ACS [5, 13].

From our study results, we found that 77.4% of those who received immediate whole body CT had positive acute findings including dissecting aortic aneurysm, Cardiac tamponade, intracranial hemorrhage, pulmonary embolism. However, there is probably no doubt that cardiac tamponade, massive hemothorax, pleural effusion, and pneumothorax could all be detected more efficiently and with little delay by other simple image modality such as bedside ultrasonography. The rate of intracranial hemorrhage detected by CT in our study was 4.3%. In other relevant literatures, the reported rate of intracranial hemorrhage in the ECMO population ranges from 10.7 to 37% [14, 15]. Immediate CT has the advantage of detecting intracranial bleeding early and may directly influence anti-thrombotic management during ECMO support.

When considering CPR related injury, 16.1% of the immediate CT group had findings on CT showing sternal or multiple rib fractures, which might be related to chest compression during CPR but no immediate intervention was required for those patients. Our findings seemed comparable to a French registry showing a 11% rate of chest skeletal fractures after CPR in OHCA patients [16]. However, in a similar study, Zotzmann et al. observed rib or sternal fractures on CT in up to 65% of their patients after ECPR [17]. Studies have shown that rib fracture is the most common injury induced by chest compression, and the prevalence may range from 13% to over 90% [18–22]. The heterogeneous results between studies might be related to differences in methodology, study population, and detection method.

In our cohort of 93 OHCA patients who received early whole-body CT, 53 (57%) showed a visible defect in myocardial enhancement of the corresponding coronary artery distribution. Among them, 88.7% had relevant findings on coronary angiography. The result might suggest that CT was reasonably sensitive and specific for the detection of AMI and may have a role in identifying patients who presents with atypical symptoms of ACS (such as in diabetics and in women) [23] or equivocal electrocardiographic / laboratory findings. Several case reports and retrospective studies have previously reported the detection of AMI on contrast-CT [24–27]. Gosalia et al. reported a total of 18 patients with a diagnosis of AMI, 15 of them had a focal decrease in left ventricular myocardial enhancement in a specific coronary distribution on contrast-enhanced CT [28]. The sensitivity of CT for detecting AMI in this study was approximately 83%, which was comparable to our present study (88.7%). However, since coronary angiography is almost always done for OHCA patients, we believe early CT for the detection of AMI would not be a justifiable indication. In our series, a large proportion of OHCA patients had received coronary angiography. The main reason was mainly because that the standard electrocardiographic or symptomatic criteria of AMI were often difficult to interpret in setting of OHCA, especially when the initial rhythm was ventricular tachycardia or ventricular fibrillation. Therefore, we have a low threshold for coronary angiography in patients with risk factors for coronary artery disease in our institution.

An interesting point that worth mentioning is that from our cohort of 136 OHCA patients, there were 43 patients who did not receive immediate CT. By reviewing the medical records, we found that 28 (65.1%) of those who did not receive immediate CT were transported to the catheterization room for coronary angiography after ECPR. This is reasonable especially when patient presented with a typical history or risk factors for coronary artery disease. Secondly, for those who did not receive immediate CT, 15 (34.9%) had delayed CT (mostly brain) later in their hospital admission. 9 had hypoxic brain injury, 2 had stroke, 1 had subdural hemorrhage (SDH) and 3 had negative findings. As there is currently no local guideline or protocol to guide clinicians as to when CT scan should be performed after ECPR in OHCA patients in our institution, so the decision is usually made at the discretion of the clinician, taking into account of various factors such as availability, patient’s clinical history, low flow duration and whether the arrest was unwitnessed / witnessed etc.

Several studies have investigated the prognostic value of brain CT in predicting neurological outcome following ECPR. Acute hypoxic brain injury may be manifested on brain CT as loss of Grey-white matter differentiation, brain swelling, or non-specific low density lesions [29]. More objective predictors such as grey-to-white matter ratio (GWR) [30], cortical sulcal effacement (SE), optic nerve sheath diameter (ONSD) [31] and Alberta Stroke Program Early Computed Tomography Score (ASPECTS) [32] have also been investigated. In our study, loss of grey-white matter differentiation and brain edema with effacement of cerebral sulci were mainly used to diagnose hypoxic brain injury, as it is less cumbersome despite being more subjective. When a CPC score of 3 ~ 5 were used as a measure for poor neurological outcome, we found that CT had a specificity of 95.5% for identifying patient with profound neurological impairment after ECPR, with a low false positive rate of only 3.7%. Logistic regression analysis also suggested a potential correlation between CT findings of hypoxic brain injury and poor neurological outcome at discharge. AHA guidelines on post resuscitation arrest care had mentioned that a marked reduction in GWR on brain CT obtained with 2 h after OHCA may be used to predict poor outcome [33]. However, our results showed that CT had a relatively poor sensitivity in predicting poor neurological outcome. This can be explained by the fact that hypoxic brain injury may not be obvious on CT when it is performed early after ECPR. Based on our clinical observation, a significant proportion of our patient with normal brain appearance on initial brain CT will have findings of hypoxic brain injury when brain CT is repeated later in the clinical course. Based on previous literature, hypoxic brain injury is usually not apparent on brain CT immediately after cardiac arrest, but features including brain swelling and loss grey-white matter will appear after three days [34]. It is therefore reasonable to recommend that OHCA patients with normal brain CT after ECPR should have their brain CT repeated at least three days later, especially when the patient shows no sign of neurological recovery after ECPR. Our findings may have important implications for clinical decision making, especially on when to withdraw the patient from ECMO support, but one must be cautious that CT should be used as the sole indicator for the prediction of neurological outcome, mainly because of unstandardized acquisition and interpretation in most centers.

Our study has limitations. First of all, we found a significantly longer LFD in patients who received immediate CT, and the reason for that might be that for those with relatively shorter LFD, immediate coronary angiography is often chosen as initial investigation to rule out potential occluded coronary vessels. This finding might indicate the presence of selection bias and could potentially overestimate or underestimate the incidence of the CT findings. The relatively small number and the heterogeneous nature of our study population may have had some impact on data analysis. In addition, our data was from a single institute and variation in regional practice might have some influence on the study findings. It is also possible that techniques and outcomes might have changed over time, since our study was carried out over an extended period from 2006 to 2019. Finally, from our data it was impossible to determine the temporality or the causal relationship of our CT findings. Therefore, though our data provide useful insight, larger prospective study is required to define the clinical role of CT after ECPR in OHCA patients.

## Conclusions

Immediate whole-body CT after ECPR in OHCA patients remains a valuable tool despite the fact that most cases of OHCA were related to ACS. It may be particularly useful when CPR-related injury or non-ACS causes of OHCA are suspected, as well as in cases where the cause of OHCA is unknown. On the contrary, routine brain CT appears to be a valuable tool in guiding anticoagulant therapy during ECMO and in aiding outcome prediction. It seems reasonably specific, with a low false positive rate and may be considered as a predictor for poor neurological outcome in OHCA following ECPR.

## Supplementary information

**Additional file 1.** CT Protocol for Patients on Veno-arterial Extracorporeal Membrane.

**Additional file 2: Figure S1.** A and B, Coronal & axial sections of contrast-enhanced computed tomography scan of the chest demonstrate regional defect in myocardial perfusion of the left ventricular wall, compatible with left main coronary lesion. C and D, Coronary angiography images of the same patient show near total occlusion of the left main coronary artery.

**Additional file 3: Figure S2.** A and B, CT of the brain shows loss of grey white matter differentiation, with diffuse swelling of the brain, compatible with severe hypoxic brain injury.

**Additional file 4: Table S1.** Outcomes of extracorporeal cardiopulmonary resuscitation in out-of-hospital cardiac arrest based on computed tomography diagnosis.

## Data Availability

The dataset used and analyzed during the current study are available from the corresponding author on reasonable request.

## Abbreviations

ACLS: Advanced cardiac life support
ACS: Acute coronary syndrome
AHA: American heart association
AMI: Acute myocardial infarction
ASPECTS: Alberta Stroke Program Early Computed Tomography Score
CCI: Charlson’s comorbidity score
CI: Confidence interval
CKD: Chronic kidney disease
COPD: Chronic obstructive pulmonary disease
CPC: Cerebral performance category
CPR: Cardiopulmonary resuscitation
CT: Computed tomography
ECMO: Extracorporeal membrane oxygenation
ECPR: Extracorporeal cardiopulmonary resuscitation
eGFR: estimated glomerular filtration rate
ERC: European resuscitation council
GWR: Grey-to-white matter ratio
IABP: Intra-aortic balloon pump
IHCA: In-hospital cardiac arrest
IQR: Interquartile range
LFD: Low flow duration
LR: Likelihood ratio
NYHA: New York Heart Association
OHCA: Out-of-hospital cardiac arrest
ONSD: Optic nerve sheath diameter
OR: Odds ratio
ROSC: Return of spontaneous circulation
SD: Standard deviation
SE: Sulcal effacement
