# Supplementary material for: Clinical experience of whole-body computed tomography as the initial evaluation tool after extracorporeal cardiopulmonary resuscitation in patients of out-of-hospital cardiac arrest
Source: Scand J Trauma Resusc Emerg Med. 2020 Jun 11;28:54. doi: 10.1186/s13049-020-00746-5 (PMC7291474; doi:10.1186/s13049-020-00746-5)
Supplement: Supplementary file 1 — Additional file 1. CT Protocol for Patients on Veno-arterial Extracorporeal Membrane. [file 13049_2020_746_MOESM1_ESM.docx]

**CT Protocol for Patients on Veno-arterial Extracorporeal Membrane**

General principles are outlined below, and the protocol may be adjusted slightly to tailor the needs of individual patient

- In our institute, 64-slice/256-slice/320-slice scanners are available. The slice thickness of the CT image is 5mm.
- For whole-body CT, scanning is performed from the brain all the way down to the perineum. Occasionally, we extend the range of scanning down to the distal thigh if any complication of the cannulation site is suspected.
- Before scanning, the length of ECMO tubing is checked to avoid dislocation of the cannulae during movement of the CT table.
- To compensate for the extra circulating blood volume within the ECMO tubing, additional contrast at a dose of 0.5ml/kg is added to the standard adult dose. Typically, the dose for IV contrast in VA-ECMO patients is around 1.5~2 ml/kg of Ultravist 370 or Omnipaque 350, up to a maximum of 150ml per scan.
- For venous-arterial (VA) ECMO, the level of ECMO support needs to be determined prior to contrast injection.
- In the context of (near) full support, IV Contrast is administered via a central venous catheter or a large peripheral intravenous cannula at a rate of 3-5ml/s. When acquiring the arterial phase images, we place the monitor plane at the level of the aortic valve in order to observe the sequential enhancement of the right ventricle, descending aorta, pulmonary artery and aortic root. Scanning is triggered manually with minimal delay as soon as the contrast arrives at the aortic root.
- In the context of partial support, it is sometimes difficult to obtain a good uniform arterial phase image due to the presence of watershed phenomenon (also known as the Harlequin phenomenon). Therefore, we need to determine the level of the watershed and inject the contrast either from above or below the watershed level, depending on the body area of interest.
- After the arterial phase, the acquisition of the venous phase images will begin following a delay of approximately 90-150 seconds
- The images are reviewed on the CT console by the radiologist and determined if any additional delayed phase images are needed (after a further delay of 3-5 minutes).
