# Supplementary figures and images for: Clinical experience of whole-body computed tomography as the initial evaluation tool after extracorporeal cardiopulmonary resuscitation in patients of out-of-hospital cardiac arrest
Source: Scand J Trauma Resusc Emerg Med. 2020 Jun 11;28:54. doi: 10.1186/s13049-020-00746-5 (PMC7291474; doi:10.1186/s13049-020-00746-5)

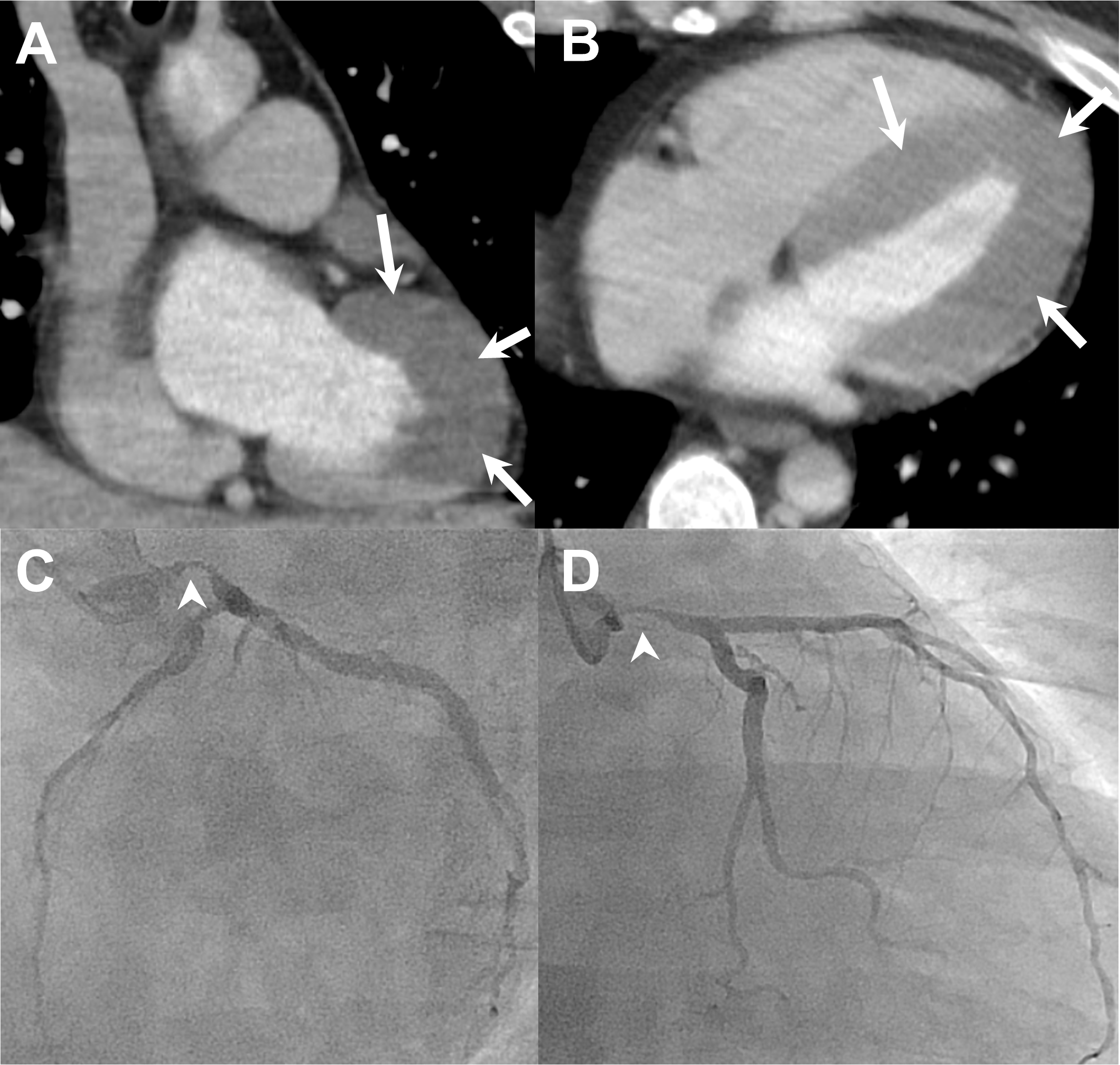

Supplement: Supplementary file 2 — Additional file 2: Figure S1. A and B, Coronal & axial sections of contrast-enhanced computed tomography scan of the chest demonstrate regional defect in myocardial perfusion of the left ventricular wall, compatible with left main coronary lesion. C and D, Coronary angiography images of the same patient show near total occlusion of the left main coronary artery. [file 13049_2020_746_MOESM2_ESM.tif]

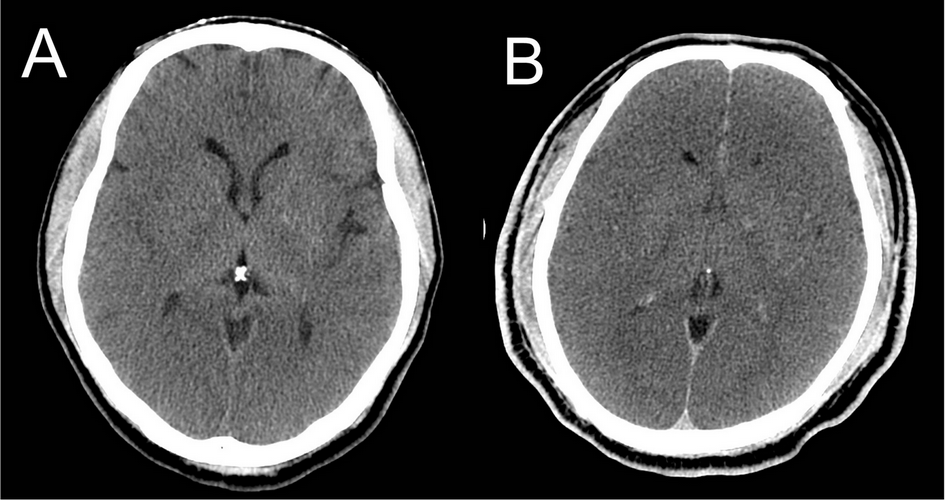

Supplement: Supplementary file 3 — Additional file 3: Figure S2. A and B, CT of the brain shows loss of grey white matter differentiation, with diffuse swelling of the brain, compatible with severe hypoxic brain injury. [file 13049_2020_746_MOESM3_ESM.tif]
