# Supplementary material for: Clinical experience of whole-body computed tomography as the initial evaluation tool after extracorporeal cardiopulmonary resuscitation in patients of out-of-hospital cardiac arrest
Source: Scand J Trauma Resusc Emerg Med. 2020 Jun 11;28:54. doi: 10.1186/s13049-020-00746-5 (PMC7291474; doi:10.1186/s13049-020-00746-5)
Supplement: Supplementary file 4 — Additional file 4: Table S1. Outcomes of extracorporeal cardiopulmonary resuscitation in out-of-hospital cardiac arrest based on computed tomography diagnosis. [file 13049_2020_746_MOESM4_ESM.docx]

**Supplemental table 1. Outcomes of extracorporeal cardiopulmonary resuscitation in out-of-hospital cardiac arrest based on computed tomography diagnosis**

| **Outcomes** | **CT (+) for AMI, n=53** | **CT (+) for Hypoxic brain injury, n=27** | **CT (+) for cerebral hemorrhage, n=4** | **CT (+) for cerebral infarction, n=4** | **CT (+) for cardiac tamponade, n=5** | **CT (+) for Dissecting aortic aneurysm, n=7** | **CT (+) for pulmonary embolism, n=5** |
| --- | --- | --- | --- | --- | --- | --- | --- |
| Ventilator days | 14.4 ± 24.4 | 12.0 ± 31.6 | 14.5 ± 19.3 | 13.0 ± 16.1 | 2.6 ± 3.2 | 3.7 ± 5.9 | 3.6 ± 3.8 |
| ECMO days | 3.9 ± 4.3 | 2.6 ± 3.0 | 3.0 ± 3.5 | 6.0 ± 6.7 | 2.6 ± 3.2 | 3.6 ± 6.0 | 3.0 ± 3.9 |
| ICU days | 12.5 ± 14.3 | 6.7 ± 13.1 | 16.0 ± 19.5 | 14.5 ± 18.9 | 2.6 ± 3.2 | 3.6 ± 5.8 | 4.2 ± 4.0 |
| Hospitalization Days | 22.2 ± 31.1 | 11.9 ± 31.6 | 39.3 ± 52.8 | 31.0 ± 51.1 | 2.6 ± 3.2 | 3.6 ± 5.8 | 8.0 ± 11.2 |
| Die on ECMO, n (%) | 25 (47.2%) | 21 (77.8%) | 2 (50.0%) | 3 (75.0%) | 5 (100%) | 7 (100%) | 4 (75%) |
| Weaned off ECMO and die, n (%) | 7 (13.2%) | 2 (7.4%) | 1 (25.0%) | 0 (0.0%) | 0 (0.0%) | 0 (0.0%) | 0 (0.0%) |
| Survival on discharge, n (%) | 21 (39.6%) | 4 (14.8%) | 1 (25.0%) | 1 (25.0%) | 0 (0.0%) | 0 (0.0%) | 1 (20%) |
| CPC 1/2 on discharge, n (%) | 14 (26.4%) | 1 (3.7%) | 0 (0.0%) | 1 (25.0%) | 0 (0.0%) | 0 (0.0%) | 1 (20%) |
| ECMO to CT time | 69.6 ± 25.3 | 70.0 ± 26.0 | 75.2 ± 21.0 | 76.2 ± 16.5 | 95.0 ± 46.0 | 79.9 ± 37.0 | 71.8 ± 11.9 |

CT (+) = CT positive, AMI= Acute myocardial infarction, ECMO= Extracorporeal membrane oxygenation, CPC= Cerebral performance category
